# Association of vitamin D deficiency and pelvic organ prolapse in postmenopausal women: a cross-sectional study

**DOI:** 10.1186/s40695-022-00078-7

**Published:** 2022-08-05

**Authors:** Mateja Legan, Matija Barbič, Joško Osredkar, Mija Blaganje

**Affiliations:** 1 Division of Gynaecology, University Medical Centre Ljubljana, Zaloška 7, 1000 Ljubljana, Slovenia; 2 Institute of Clinical Chemistry and Clinical Biochemistry, Zaloška 7, 1000 Ljubljana, Slovenia

**Keywords:** Pelvic organ prolapse, POP-Q classification, Vitamin D, Postmenopause

## Abstract

**Background:**

Vitamin D is vital for skeletal integrity as well as optimal muscle work. High incidence and prevalence of vitamin D deficiency as well as pelvic organ prolapse are found in postmenopausal women, thus raising the question of whether the entities could be related.

**Methods:**

We compared 50 postmenopausal women aged 50 to 75 years with pelvic organ prolapse (POP) with 48 women of same age without POP. The clinical assessment of the disorder was performed using the Pelvic Organ Prolapse Quantification system (POP-Q). An anamnestic questionnaire was filled out by the participants on their anthropometric data, life habits, reproductive history, previous and actual diseases. A blood sample was collected for determination of 25-OH-vitamin D as well as calcium and phosphorus concentrations.

**Results:**

The group with POP and the control group were comparable in body mass index, physical activity, life habits and general health, but differed significantly in parity (being higher in POP) and vitamin D blood level concentrations, being lower in POP patients. A significantly higher prevalence of vitamin D deficiency (25-OH-vitamin D < 50 nmol/l) was found in the POP group compared to controls. Taking into account the confounding variables the logistic regression model confirmed the significant role of vitamin D for POP.

**Conclusions:**

Vitamin D deficiency might be an important systemic factor associated to pelvic organ prolapse. The determination of vitamin D levels in postmenopausal women and replenishing its deficiency might also be of importance for the pelvic floor.

## Background

Pelvic organ prolapse (POP) is affecting millions of women worldwide. It is caused by the weakening of the pelvic floor supportive tissue and occurs independently or coexists with other pelvic floor dysfunction, with a lifetime risk for surgery as high as 20% [1] and with a substantial reoperation rate due to recurrence [2]. The supportive soft tissues within the pelvic floor are a combination of muscles, fascias, and ligaments working together to keep the pelvic organs in place in a highly dynamic environment, to provide support and resist deformations [3]. Evidence suggests that weakness of the supportive tissues, either at the systemic or local level, may predispose to POP, suggesting that underlying supportive tissues of the pelvic floor are made of weak components and these components are more likely to fail or stretch leading to POP [4]. Various factors may affect the functional capacity of the pelvic floor. Knowing the prevalence of vitamin D deficiency, its recently observed potentially protective actions on tendons, ligaments and connective tissue [5] and its known effect on the longitudinal and striated muscle [6–8], the role of this factor became a subject of observation in a few clinical studies in recent years, already bringing some promising, but inconsistent results [9–11].

In the last two decades, epidemiological increase of vitamin D deficiency in the common population has been confirmed. It has been estimated that 20–80% of US, Canadian and European elderly men and women are vitamin D deficient [12, 13]. Being a major factor in maintaining calcium and phosphorus homeostasis, in addition to calciotropic hormones, vitamin D is involved in bone integrity. Its active form 1,25(OH)_2_D_3_ exerts its biological effects through vitamin D receptors (VDRs). These receptors are present also in the smooth and skeletal muscles [6, 14]. Through them vitamin D has impact on the proper functioning of skeletal muscles by regulating calcium homeostasis to affect muscle contractility and by maintaining muscle cell environment against inflammation [7]. In case of deficiency, a smaller number of type 2 muscle fibres are present in the muscle [8], and muscle weakness is obvious not only in cases of overt deficiency, but also of insufficiency. In POP, fibrosis and the degradation of the connective tissue in the vaginal wall predominate and the aggravation of degenerative changes in the connective tissue lead to its progression [15]. Women with POP have more type III collagen, than women without the disease; type III collagen being predominant in tissues that require increased distensability and elasticity and its increase manifesting itself in vaginal extensibility [4].

We wanted to understand how important vitamin D deficiency/insufficiency is in the Slovenian female population suffering from POP. We expected it to be important in addition to the known factors influencing the pelvic floor, such as parity, exercise and chronic coughing. We conducted a prospective study on postmenopausal women with objective morphological evaluation of the pelvic floor status using the POP-Q system [16].

## Methods

### Patients

One hundred and two consecutive patients all attending the Gynaecological Outpatient Department at the University Medical Centre Ljubljana aged 50 to 74 years were assessed for either POP (test group) or other unrelated condition (control group). The exclusion criteria were conditions affecting the muscle function (asthma/chronic obstructive pulmonary disease, chronic cough, muscle or connective tissue disease, nerve disease, i.e. multiple sclerosis). To account for real current vitamin D status of the women, treated osteoporosis at the time of the research and supplementation of more than 400 IU of vitamin D daily were also criteria of exclusion.Two participants were excluded from the analyses due to high-dose supplemental vitamin D treatment of osteoporosis, one from test and 1 from control group. Also, one patient was excluded from the control group due to multiple sclerosis, identified only after later review of the anamnestic questionaire. One patient was too young to include in the study and thus excluded. The final number of participants was 98, among them 50 POP patients and 48 controls. The study was approved by the National Committee for Medical Ethics and written informed consent was obtained from all participants.

### Protocol

During two consecutive years of sun-deprived winter months from November to April, 50 women with POP and 48 controls were recruited. All participants completed anamnestic questionnaire on anthropometric data, previous and current disease, parity and life habits (physical activity, coffee intake and smoking). The body mass index was calculated from the data on weight and height. A blood sample was taken in the morning hours for detecting the value of 25-OH-D_3_ in the serum; serum calcium and phosphorus were also routinely measured. One of the gynaecologists (M.B. or M. Ba.) performed a gynaecological examination using the Pelvic Organ Prolapse Quantification System (POP-Q) as an objective, site-specific examination system describing and quantifying the location of different points along the vaginal wall for staging the degree of POP [16]. The control group of age- and weight-matched women had no POP.

### Laboratory meassurements

Vitamin D – more precisely 25-OH-D_3—_was determined from two batches (April 2018 and 2019), minimzing interassay variability. The sera were frozen until the time of analysis. Serum 25-OH-D_3_ (in the following text 25-OH-D, also vitamin D) levels were measured using a direct competitive chemiluminescence immunoassay (CLIA). During the first incubation, 25-OH-D is dissociated from its binding protein and binds to the specific antibody in the solid phase. After 10 min the tracer (vitamin D linked to an isoluminol derivative) is added. After second 10-min incubation, the unbound material is removed with a wash cycle. Subsequently, the starter reagents are added to initiate a flash chemiluminescent reaction. The light signal is measured by a photo multiplier as relative light units and is inversely proportional to the concentration of 25-OH-D present in calibrators, controls and samples. The LIASON 25-OH-D assay has an analytical sensitivity of 10 nmol/l. Intraassay precision/coefficient of variability (CV) is 2 to 4% and interassay CV is 7%.

Twenty-five-hydroxy-vitamin D values of < 50 nmol/l are considered to be deficient, values < 25–30 nmol/l are associated with osteomalacia. However, values 50–74 nmol/l are considered to be insufficient. Values 75–125 nmol/l are considered normal.

### Statistical analyses

Statistical analyses were performed using SPSS version 25 statistical program. Descriptive statistics (mean, standard deviation, median) were calculated for continuous variables. After prior testing for normal distribution, the Student’s t-test for indenpendent samples was applied to compare differences between test and control groups. For categorical variables, frequencies were obtained and Pearson Chi-squared test was applied. Different bivariate correlations (Pearson coefficients as well as Spearman – rank coefficients) were calculated between chosen pairs of variables. A multiple logistic regression analysis was used for assessing the effect of different independent variables (continuous as well as categorical) to POP/controls. A *p*-value < 0.05 was considered statistically significant in all the calculations.

## Results

Ninety-eight postmenopausal women aged from 49 to 75 (mean age 60.7 years, median 60.0 years) were recruited, 50 of them suffered of POP; the other 48 were weight-matched controls of the same age span. All women were of good health. Thirteen patients had well controlled hypertension, as did 11 controls (*p* = 0.72). Five patients and 1 control had type 2 diabetes (*p* = 0.09), on oral medication without any significant diabetic complications. 6 participants had osteoporosis and were treated in the past. Personal anthropometric characteristics of our patients are shown in Table 1. Due to a wide age range of partcipants a statistically important difference resulted in the mean age between the test and control group (62.6 ± 9.3 vs. 58.7 ± 7.2 years). Participants had comparable BMI, age at menarche and age at menopause.

**Table 1:** Personal characteristics of participants

	**POP patients (No = 50)**	**Controls** **(No = 48)**	***P* -value**
**Age (yrs ± SD)**	62.6 ± 9.3	58.7 ± 7.2	**0.024**
**BMI (kg/m**^2^ **± SD)**	27.6 ± 3.8	27.1 ± 5.2	0.65
**Menarche (age ± SD)**	13.4 ± 1.6	12.9 ± 1.7	0.17
**Menopause (age ± SD)**	50.5 ± 3.6	50.1 ± 3.2	0.53

Mean parity in the test group was 2.3 (± 0.8) vs. 1.8 (± 0.7) in the control group (*p* < 0.01). Because the distribution of parity, vaginal deliveries and caeserean sections was not normal, we used nonparametric tests to compare POP and control groups (Table 2).

**Table 2:** More personal characteristics of participants

	**POP patients** **(No.)**	**Controls** **(No.)**	***P* -value**
**Parity** **0/1/2/3/4/5**^a^	0/5/31/12/0/2	1/14/27/5/1/0	**0.04**
**Vaginal deliveries** **0/1/2/3/4/5**^b^	0/5/32/11/0/2	5/11/26/5/1/0	**0.02**
**Caesarean sections** **0/1/2**^c^	49/1/0	4/3/1	0.33

^a^ 0 delivery/1 delivery/2 deliveries/3deliveries/4 deliveries/5 deliveries;

^b^ 0 vaginal delivery/1 vaginal delivery/2 vaginal deliveries/3 vaginal deliveries/4 vaginal deliveries/5 vaginal deliveries,

^c^0 caesarean section/1 caesarean section/2 caesarean sections

Table 3 includes laboratory results of our participants: measurements of 25-OH-D, serum Ca and P. Groups differed significantly in vitamin D levels: the test group had significantly lower blood vitamin D levels (mean 25-OH-D level in POP patients was 42.9 ± 18.8 vs. 50.9 ± 21.1 in controls, *p* = 0.049). We evaluated the clinical significance of vitamin D levels and checked for vitamin D deficiency in our participants. Indeed, POP patients had a significantly higher prevalence of vitamin D deficiency, as shown in Table 4. There were 33 (66.0%) patients with vitamin D deficiency compared to 21 (43.8%) in controls. In the test group there were also 15 (30.0%) vitamin D insufficient subjects and only 2 patients (4%) with normal vitamin D levels compared to 20 vitamin D insufficient controls (41.6%) and 7 controls (14.6%) with normal vitamin D levels (< 0.05).

**Table 3:** Results of vitamin D determination and bio-chemical measurements in participants

	**POP patients (No = 50)**	**Controls** **(No = 48)**	***P* -value**
**25-OH-D (nmol/l ± SD)**	42.9 ± 18.8	50.9 ± 21.1	**0.049**
**Ca (mmol/l ± SD)**	2.32 ± 0.13	2.33 ± 0.1	0.69
**P (mmol/l ± SD)**	1.13 ± 0.23	1.13 ± 0.18	0.98

^Values for Ca and P were all in a normal range^

**Table 4:** Prevalence of vitamin D deficiency in POP patients vs. controls

	**POP patients** **No. (%)**	**Controls** **No. (%)**
**Vitamin D deficiency—YES**	33 (66.0%)	21 (43.8%)
**Vitamin D deficiency—NO**	17 (34.0%)	27 (56.2%)
**Total**	50	48

Chi^2^ (1) = 4.90, ***p* = 0.027**

The results of POP-Q test showed that 15 POP patients had a stage 2 prolapse, 32 a stage 3 prolapse and 3 patients a stage 4 prolapse. The control group included 31 participants without prolapse as well as 17 participants with stage 1 asymptomatic physiological prolapse considered normal in women with parity history and evaluated for a condition other than pelvic floor disfunction (PFD) [17]. Participants did not differ significantly in the incidence of vitamin D deficiency at different prolapse stages (*p* = 0.21), but bivariate analyses between degree of POP (0 to 4) and vitamin D levels showed significant moderately high negative corelation (*r* = -0.24, *p* = 0.018, *n* = 98).

The results of the anamnestic questionnaire regarding personal habits, physical activity, pelvic floor muscle training, sexual activity and hormone replacement therapy are shown in Table 5. Physical activity was evaluated (from sedentary lifestyle to intensive physical excercise) and no important difference between the patients and controls were observed. Dividing the participants into two groups regarding sufficient and insufficient physical activity (Table 5) did not indicate a significant difference between patients and controls, either. Smoking habits were comparable in both groups. However, more patients than controls were taking hormone replacement therapy, almost reaching statistical significance (*p* = 0.06).

**Table 5:** Results of anamnestic questionary on life facts and habits

	**POP patients** **YES/NO (No.)**	**Controls** **YES/NO (No.)**	***P*-value**
**Physical exercising**	28/22	26/22	0.85
**Sexual intercourse**	16/14	21/27	0.23
**Pelvic floor muscle training**	31/19	28/20	0.71
**Hormone replacement therapy**	6/44	1/47	0.06
**Smoking**	9/41	7/41	0.64
**Coffee**	6/35**//**9^a^	1/40**//**7^a^	0.07

^a^ high intake/moderate intake (3 coffees or less)//no coffee

**Fig. 1:**
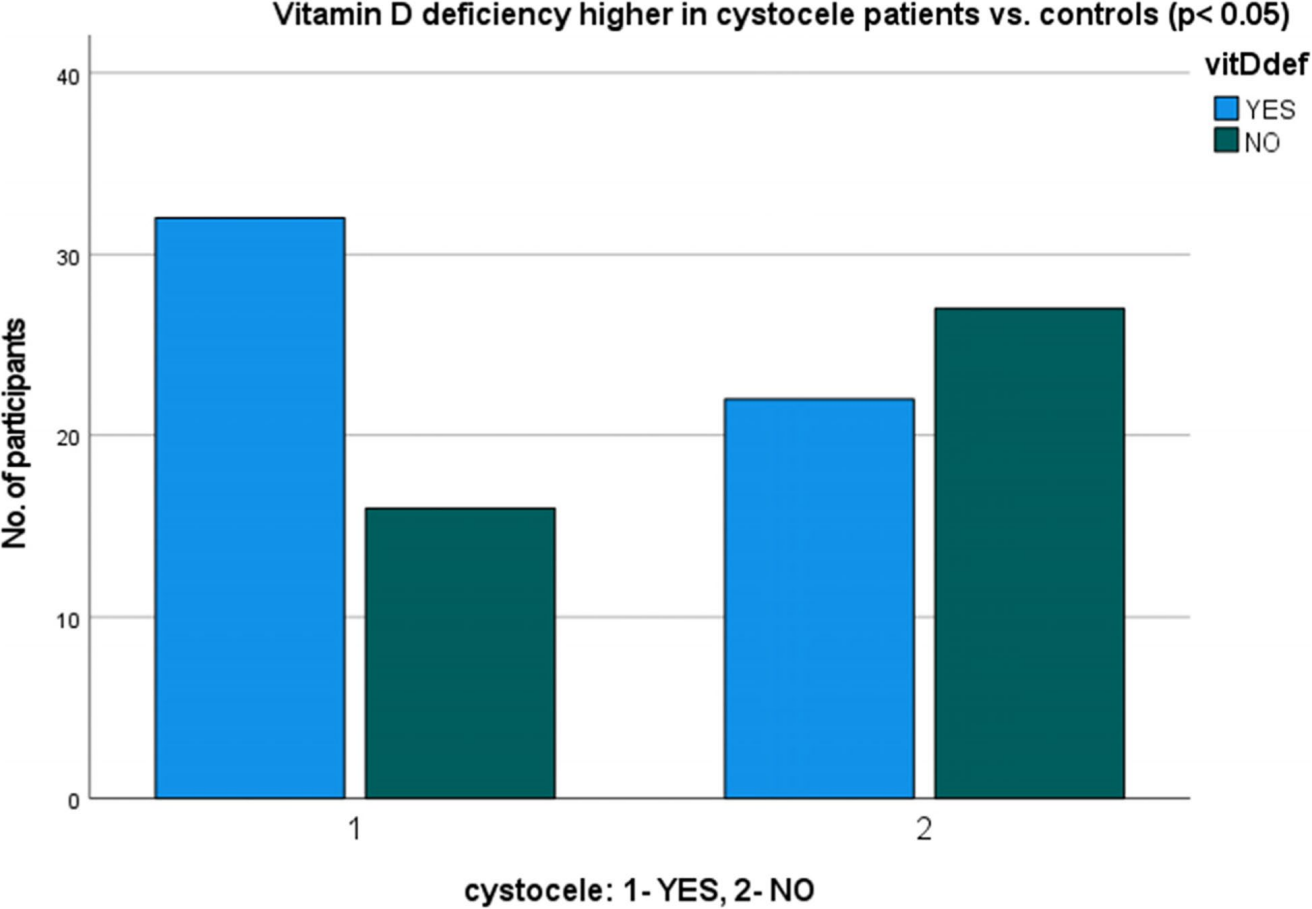
Prevalence of vitamin D deficiency in cystocele patients

**Fig. 2:**
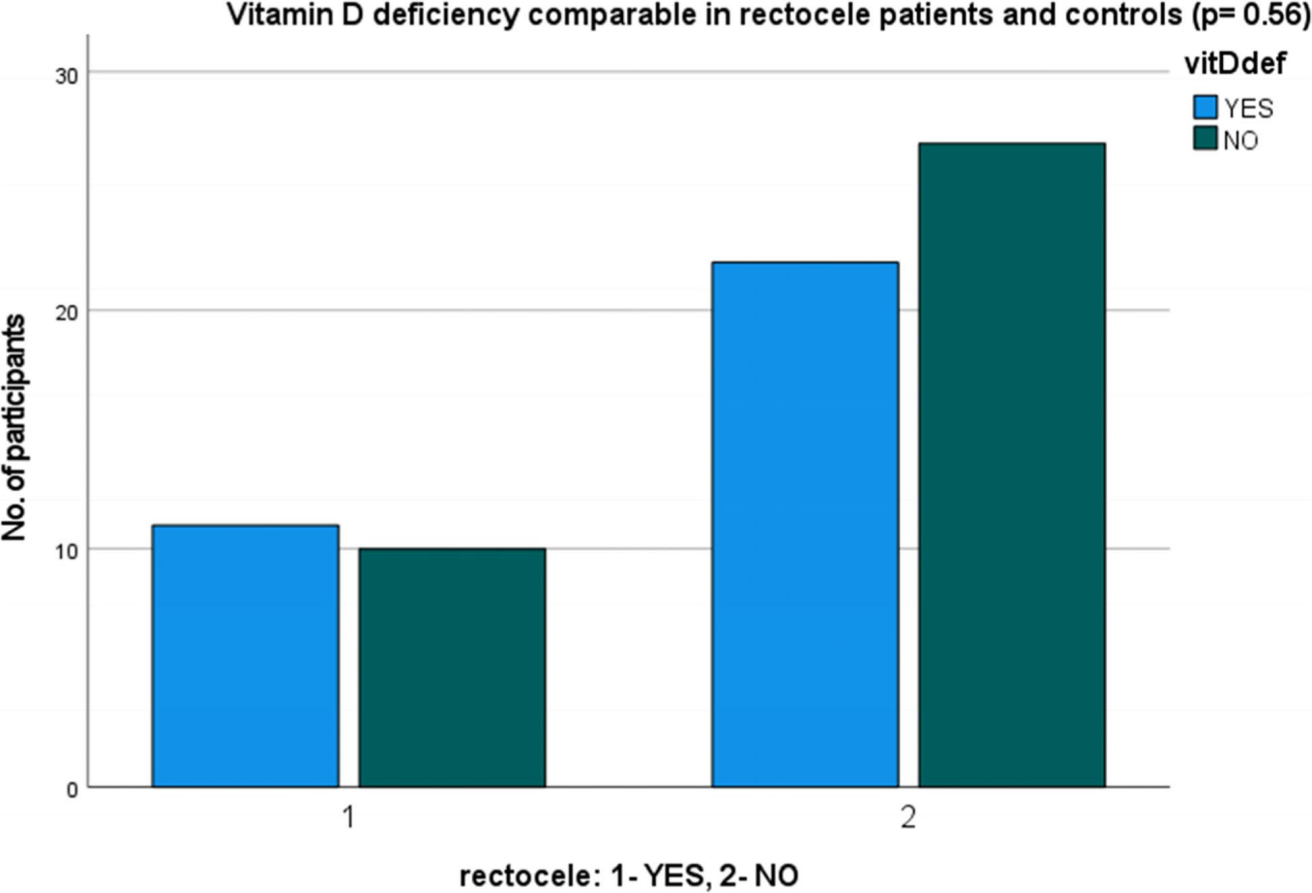
Prevalence of vitamin D deficiency in rectocele patients

Regarding the pattern of POP, 28 patients had cystocele, 20 patients had a combination of cystocele and rectocele, but 1 patient had rectocele only. In 1 patient with POP there was an isolated apical prolapse without cysto- or rectocele. Patients with a cystocele were more prone to vitamin D deficiency (*p* < 0.05), but patients with a rectocele (*p* = 0.56) were not (Figs. 1 and 2).

To assess the role of predictors in POP a multiple logistic regression model was analyzed (Table 6). According to it, vitamin D levels and parity remain significant predictors for POP. None of the other possible factors, including age, were significant.

**Table 6:** Statistics of logistic regression for predictors: vitamin D levels, age, parity, pelvic floor muscle training (PFMT), sexual activity, physical exercising, BMI, and response variable POP (yes/no)

	**B**	**S.E**	**Wald**	**df**	**OR**	**95% C.I** **lower**	**95% C.I** **upper**	***P*-value**
**25-OH-D**	-0.026	0.012	4.297	1	0.975	0.951	0.999	**0.038**
**Age**	0.057	0.032	3.100	1	1.058	0.994	1.127	0.078
**Parity**	0.836	0.365	5.246	1	2.306	1.128	4.714	**0.022**
**PFMT**	-0.379	0.496	0.584	1	0.685	0.259	1.810	0.445
**Sexual activity**	-0.263	0.509	0.267	1	0.769	0.283	2.085	0.605
**Physical exercising**	0.131	0.510	0.066	1	1.140	0.420	3.099	0.797
**Coffee**	0.300	0.524	0.328	1	1.350	0.484	3.767	0.567
**BMI**	-0.009	0.053	0.025	1	0.992	0.893	1.101	0.873

The logistic regression model was significant (*p* = 0.027), with high p level of Hosmer–Lemeshow test (*p* = 0.27) that shows a very good fit of the model. The model explaines 70.7% of cases

## Discussion

This study demonstrates that vitamin D deficiency is an independent and important factor in POP in a female postmenopausal population as hypothesised. Significantly lower vitamin D levels were found in POP group and a significant prevalence of vitamin D deficiency was found in patients with POP compared to controls. A significant correlation was found between vitamin D deficiency and cystocele, but not vitamin D deficiency and rectocele. Among the possible factors affecting the pelvic floor, parity and vaginal deliveries showed significant importance as expected, since caesarian section appears to be protective against POP.

Our results regarding vitamin D are consistent with some previous publications. Badalian and colleagues [9] found that higher vitamin D levels are associated with decreased risk of pelvic floor dysfunction in women. The study included women of all ages; PFD was evaluated by anamnestic questionnaire. Based on a cohort of 349 participants Parker-Autry et al. [10] concluded that insufficient vitamin D was associated with increased colorectal symptoms and greater impact of urinary incontinence on the quality of life. However, no significant correlation was found between lower urinary symptoms and vitamin D deficiency in the research by Aydogmus and coworkers [11], but they stressed the necessity of further investigation of pelvic floor integrity and functions. Interestingly, the study of Vaughan and colleagues [18] showed a potential association between vitamin D and the development of urinary incontinence in a racially diverse cohort of older men and women. A very recent study by Kaur et al. [19] confirms that vitamin D levels were associated with a decreased risk of pelvic floor disorders in geriatric females. In their research, Hyung Ahn et al. [20] examined vitamin D levels and performed a VDR genotype analysis. The presence of a certain sequence of the vitamin D receptor (VDR) polymorphism (Apal and Bsmi) was associated with PFD in vitamin D deficient subjects.

To avoid seasonal variations in vitamin D concentrations we opted for the determination of 25-OH-D during winter time. Our study and control groups were meant to be of comparable age and they were weight-matched, both being important factors in vitamin D concentrations. Although the mean age of patients and controls was 62.6 years and 58.7 years, which was statistically significantly different (*p* < 0.05), this difference was not unexpected given since our study age span included postmenopausal women between 50 and 75 years. Of the most importance is that our participants were weight-matched, because of the known significant inverse correlation between vitamin D levels and BMI [21, 22]. Since vitamin D is soluble in body fat, the reduced bioavailability of vitamin D results in obesity [23].

A strength of our study is that both of our groups were comparable in behavioural factors impacting the pelvic floor: physical and sexual activity, habits (coffee and smoking) and pelvic floor muscle training. Considering reproductive parameters that might influence pelvic floor, parity—being a known risk factor for POP, differed among the two groups (*p* < 0.05), whereas caesarean section did not. It is known that the odds for POP increase tenfold with single vaginal birth and additional vaginal births cause no significant further increase in risk for POP [24]. However, logistic regression analysis in our study revealed a significant association with vitamin D even after adjustment for parity and other predictors for POP. Vitamin D and parity were significant predictors in the logistic regression model for POP, being predictive in opposite directions: higher vitamin D levels – lower risk for POP, higher parity – higher risk for POP.

PFD-POP was objectified using the POP-Q examination system in the present study. The numerical quantification of the disorder yielded a homogeneous recruitment and classification of participants, where subjective factors were minimized. Previous investigations were not based on POP-Q assessment [9, 10], except for the study of Kaur and coworkers [19], and Adognymus and coworkers [11]. However, none of these studies assessed possible differences in risk among the different compartments of prolapse.

In our study cystocele was associated with vitamin D deficiency, whereas rectocele was not. This was not necessarily a surprise taking into account that abnormal pelvic floor muscles are observed more often in women with anterior prolapse than with posterior prolapse [25], and that anterior prolapse in a great number of cases is also linked to the descent of the apical vaginal support, hence sharing a more complex mechanism in terms of different pelvic support structures involved [26]. Additionally, women with cystocele have the most compliant anterior and posterior vaginal wall support systems when compared to women with rectocele and normal support [4]. As vitamin D plays a role in different support tissues (i.e. striated and smooth muscles, tendons, fascias and connective tissue), we speculate that the likelihood of clinical manifestation of its deficiency is more likely to be pronounced in pelvic floor conditions where many of those support tissues and structures are involved.

We strongly believe that the results of our study are clinically important: for vitamin D deficiency/insufficiency, low cost and effective vitamin D supplementation treatment is available. In an Indian study [19], the regression of symptoms of urinary incontinence as well as the positive effect on the symptoms of PFD were observed after 6 months of treatment. Further clinical studies on the effect of supplementation are needed.

## Conclusions

Our study revealed a significant association between vitamin D levels and POP in postmenopausal women. We believe that vitamin D deficiency might be an important associated systemic factor in POP in the postmenopausal female population. The anterior vaginal wall seems more susceptible for vitamin D deficiency. Educating women and professionals about the importance of vitamin D supplementation (in case of its deficiency or insufficiency) should be an important part of a conservative approach to the prevention and treatment of POP.

## Data Availability

The datasets used and/or analysed during the current study are available from the corresponding author on reasonable request.

## Abbreviations

POP: Pelvic organ prolapse
POP-Q: Pelvic Organ Prolapse Quantification system
VDR: Vitamin D receptor
PFD: Pelvic floor dysfunction
PFMT: Pelvic floor muscle training
